# Advances in the prevention and treatment of breast cancer-related lymphedema

**DOI:** 10.1007/s10549-023-06947-7

**Published:** 2023-04-27

**Authors:** Paula M. C. Donahue, Adrien MacKenzie, Aleksandra Filipovic, Louise Koelmeyer

**Affiliations:** 1 Physical Medicine and Rehabilitation, Vanderbilt University Medical Center, 2201 21St Children’s Way, Suite 1218, Nashville, TN 37212 USA; 2 Dayani Center for Health and Wellness, Vanderbilt University Medical Center, Nashville, TN USA; 3 Osher Center for Integrative Health, Vanderbilt University Medical Center, Nashville, TN USA; 4 PureTech Health, London, UK; 5 Faculty of Medicine, Health, and Human Sciences, Australian Lymphoedema Education, Research, and Treatment (ALERT), Macquarie University, Sydney, Australia

**Keywords:** Breast cancer, Breast cancer lymphedema, Indocyanine green, Lymphedema, Manual lymphatic drainage, Optical imaging

## Abstract

**Purpose:**

Breast cancer-related lymphedema (BCRL) represents a lifelong risk for breast cancer survivors and once acquired becomes a lifelong burden. This review summarizes current BCRL prevention and treatment strategies.

**Findings:**

Risk factors for BCRL have been extensively studied and their identification has affected breast cancer treatment practice, with sentinel lymph node removal now standard of care for patients with early stage breast cancer without sentinel lymph node metastases. Early surveillance and timely management aim to reduce BCRL incidence and progression, and are further facilitated by patient education, which many breast cancer survivors report not having adequately received. Surgical approaches to BCRL prevention include axillary reverse mapping, lymphatic microsurgical preventative healing (LYMPHA) and Simplified LYMPHA (SLYMPHA). Complete decongestive therapy (CDT) remains the standard of care for patients with BCRL. Among CDT components, facilitating manual lymphatic drainage (MLD) using indocyanine green fluorescence lymphography has been proposed. Intermittent pneumatic compression, nonpneumatic active compression devices, and low-level laser therapy appear promising in lymphedema management. Reconstructive microsurgical techniques such as lymphovenous anastomosis and vascular lymph node transfer are growing surgical considerations for patients as well as liposuction-based procedures for addressing fatty fibrosis formation from chronic lymphedema. Long-term self-management adherence remains problematic, and lack of diagnosis and measurement consensus precludes a comparison of outcomes. Currently, no pharmacological approaches have proven successful.

**Conclusion:**

Progress in prevention and treatment of BCRL continues, requiring advances in early diagnosis, patient education, expert consensus and novel treatments designed for lymphatic rehabilitation following insults.

**Supplementary Information:**

The online version contains supplementary material available at 10.1007/s10549-023-06947-7.

## Introduction

Breast cancer-related lymphedema (BCRL) is one of the most feared complications of breast cancer [1, 2]. It represents a lifelong burden for many and a lifelong risk for nearly all breast cancer survivors. It can neither be cured nor easily concealed in advanced stages [3]. Its long-term burden extends beyond considerable symptoms (e.g., arm swelling, pain, limited function necessitating compensatory movement strategies) [4], to significantly impact quality-of-life (QoL), psychosocial interactions, and emotional wellbeing [5, 6], as well as cause substantial financial burdens to patients, caregivers, payers and society [7].

There is no single tool to assess BCRL, but various objective tools and more subjective clinical examination. Lack of standardized methods and protocols for assessing lymphedema has been problematic for decades limiting understanding of BCRL incidence and treatment outcomes. Table 1 shows frequently reported objective BCRL assessment methods. Patient-reported symptoms also have diagnostic value [8]. Clinical assessment methods used are typically institution- and equipment-dependent. Diagnostic threshold values may differ for a given method. BCRL prevalence estimates therefore vary widely. With an estimated 3.8 million breast cancer survivors currently in the U.S. [9], the number of patients affected by BCRL likely approaches one million. As better treatment methods extend survival in breast cancer, BCRL will represent an increasingly important consideration where identifying an accurate and reproducible tool that is readily accessible would have a momentous impact on BCRL management and treatment [10].

**Table 1:**
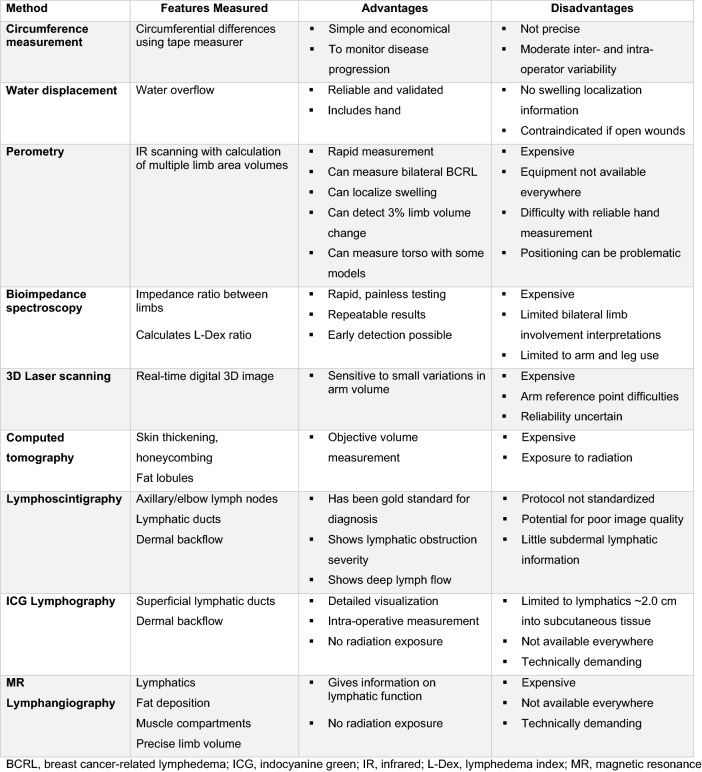
Objective diagnostic methods used in BCRL. Adapted from Pappalardo et al. [93]

## BCRL prevention

Risk factors for BCRL have been extensively reviewed [11–21] (Online Resource 1: Supplementary Table 1, Supplementary Table 2). Lymphadenectomy is the primary treatment-related risk factor for BCRL in patients with breast cancer undergoing surgery. A higher number of lymph nodes dissected is associated with increased risk [11], as is axillary (ALND) versus sentinel lymph node dissection (SLND). For example, a large, long-term study of patients with invasive breast cancer reported cumulative BCRL rates of 24.9% and 8% in the axial lymph node alone and sentinel lymph node alone cohorts, respectively [22]. Mastectomy has been associated with a significantly higher BCRL risk than lumpectomy [15], and evidence suggests that immediate post-mastectomy breast reconstruction lowers BCRL risk [16, 23, 24]. Identifying these treatment-related risk factors has affected standard surgical practice, with SLND now the standard of care for women with early stage breast cancer, and ALND contraindicated in those without sentinel lymph node metastasis [25].

Radiotherapy is also associated with increased BCRL risk [26]. A randomized study of women treated with breast-conserving surgery and adjuvant systemic therapy found that patients treated with regional nodal irradiation had a higher rate of lymphedema (8.4% vs. 4.5%; *P* = 0.001) at 9.5 years than patients not receiving it [27] (see Table 2).

**Table 2:** Recent interventional clinical trials of pharmacological agents for the treatment of breast cancer-related lymphedema

Trial	Agent	Phase	*N*	Design	Primary outcome	Results
NCT02257970 [136]	Ketoprofen	4	89	Open label exploratory, then randomized, double-blind, placebo-controlled trial of pts with arm or leg LE	Exploratory Phase: Derm. score Randomized Phase: Skin thickness	Exploratory Phase: Score improvement − 3.4 (*p* < 0.0001) Randomized Phase: Skin thickness reduced (*P* = 0.01), but no change in limb volume
NCT04243837 [137]	LYT-100	1/2	100	Part 1: Dose escalation Part 2: Food effect study Part 3: Randomized, double-blind, placebo-controlled trial in pts with Stage 1 or 2 BCRL	Part 1: safety, MTD Part 2: PK, food effect Part 3: Efficacy signals	Part 1: well tolerated, MTD not reached at 100–1000 mg BID Part 2: 19% lower exposure in fed vs. fasting Part 3: Results not yet reported
NCT02994771 [138]	Lymfactin®	1	15	Single-arm trial of adenoviral VEGF-C combined with VLNT in pts with BCRL	Safety	Well tolerated, no DLT at maximum dose
NCT03658967 [139]	Lymfactin®	2	39	Double-blind, randomized, placebo-controlled trial of adenoviral VEGF-C combined with VLNT in pts with BCRL	Arm volume; Lymphatic flow by lymphoscintigraphy; QoL per LQOLI	Company press release states that results were "inconclusive" [140]
NCT04390685	Tacrolimus, topical	1/2	60	Single-arm study of tacrolimus ointment following ALND for BCRL prevention	Arm volume change by water displacement	Not yet reported
NCT04541290	Tacrolimus, topical	1/2	20	Single-arm study of tacrolimus ointment in pts with existing BCRL	Arm volume change by water displacement	Not yet reported
NCT02494206 [141]	QBX258	NS	9	Single-arm study of anti-IL4/ anti-IL13 blockade in Stage 1 or 2 BCRL	Arm volume change by perometry	Arm volume significantly *increased* relative to baseline (*P* = 0.046)

*ALND* axillary lymph node dissection, *BCRL* breast cancer-related lymphedema, *BID* twice-daily, *LE* lymphedema, *LQLOLI* lymphedema quality of life inventory, *MTD* maximum tolerated dose, *NS* not specified, *QoL* quality of life, *VEGF-C* vascular endothelial growth factor C, *VLNT* vascularized lymph node transfer

Among proposed non-treatment-related, independent risk factors for BCRL are age [28], body mass index (BMI) at baseline [11, 20, 29], genetic factors [30], post-operative infection [31], race or ethnicity [32], and the presence of subclinical edema [33].

### Non-surgical approaches to prevention

Early surveillance with timely intervention reduces both BCRL incidence and severity [34]. A retrospective study of breast cancer survivors compared those who had begun bioimpedance spectroscopy (BIS) monitoring pre-surgery or within 90 days post-surgery with a cohort for which BIS monitoring began later (median 2.1 years) [35]. Significantly more women in the latter group were diagnosed with BCRL (any grade, 39% vs. 14%; *P* < 0.001) and BCRL severity was also higher (stage II-III, 24% vs. 4%). Prevention benefits may depend on the BCRL assessment method employed, with BIS providing more precise identification of patients more likely to benefit from early compression intervention than tape measurement in one recent study [36]. Serial near-infrared fluorescence lymphatic imaging (NIRF-LI) was associated with 83% and 86% positive and negative BCRL predictivity, respectively [37]. BCRL dermal backflow often appeared months before arm swelling, enabling earlier recognition of lymphatic dysfunction to triage for earlier treatment.

Other studies have also associated early surveillance combined with timely intervention for sub-clinical lymphedema with low rates of progression to clinical BCRL [38–42]. Early interventions include use of compression sleeves and manual lymph drainage (MLD) [43, 44]. In one prospective study of patients undergoing ALND at high risk for BCRL, regular BIS assessments at 3–6 month intervals were followed by short-term physical therapy, compression garments, and lymphedema education for those with sub-clinical lymphedema [38]. At a median 19-month follow-up, the incidence of clinical BCRL was a remarkably low 4.4%. Another study of structured BIS surveillance and early intervention reported a 3% rate of BCRL (median 24-month follow-up) [39]. In a randomized study, prophylactic compression sleeves usage significantly reduced arm swelling incidence (HR 0.61; *P* = 0.004) relative to the control group as measured by BIS among women undergoing ALND [44]. This growing body of evidence for BCRL surveillance impact underscores the critical need for elevating basic surveillance model requirements across the U.S. for triage to basic early intervention.

Patient education is an important component of BCRL prevention. Given the benefits of early BCRL treatment and in light of evidence that patient-reported arm symptoms (e.g., clothing or jewelry tightness, arm heaviness) may be prodromal [45–47], all breast cancer patients should know the importance of contacting their healthcare providers immediately should such symptoms arise [48–50]. Since cellulitis may act as a trigger for BCRL, patients should also be cautioned to avoid infections [49]. Although pretreatment lymphedema education is recommended to reduce BCRL incidence [51, 52], many patients report never having received this information [52]. Efforts must be made to ensure individualized, patient-centric education—with touchpoints throughout their cancer care—is being provided and retained by the patient. Of note, an international consensus for preventive intervention for BCRL was recently published, which provides recommendations to assist in clinical guidelines development [53]. The recommendation of high consensus involved the critical importance of adequate patient education about lymphedema, ensuring the patient understands the information and is empowered to take an active approach.

The Prospective Surveillance Model (PSM) is a comprehensive approach to survivorship healthcare for women with breast cancer [54, 55]. It provides time points for assessments and education from diagnosis through long-term survivorship, emphasizing identification and management of impairments (including BCRL) and health-promoting behaviors. An analysis estimated the cost to manage early stage BCRL per patient year using PSM at $636 and the cost to manage late-stage BCRL at $3125 per patient year, making PSM attractive from a health economics standpoint [56]. The feasibility of PSM for BCRL prevention in real-world clinical practice has been demonstrated [57, 58]. BIS monitoring with portable equipment during in-home visits may also be a viable component of the PSM, particularly for patients living far from large treatment centers and/or at high BCRL risk [59].

### Surgical approaches to prevention

Axillary reverse mapping (ARM) is a technique for identifying and sparing arm lymphatic drainage in patients undergoing ALND or SLND, aimed to minimize lymphedema risk [60]. Injecting blue or fluorescent dye into the arm allows visual differentiation of arm lymphatics from technetium-labeled breast lymphatics, and consequently their preservation during dissection. In a large, prospective study of ARM, 26-month lymphedema rates (increased water volume displacement ≥ 20%) were only 0.8% and 6.5% for patients who underwent SLND and ALND, respectively [61]. In some cases, however, crossover between the lymphatics from breast and arm has been noted, and metastatic disease may be present in ARM nodes. In addition, not all ARM nodes can be identified [62, 63].

Constructing lymphatic-venous anastomoses (LVAs) is a growing approach to treating secondary lymphedema. In Lymphatic Microsurgical Preventative Healing (LYMPHA), LVAs are used for primary prevention of arm lymphedema at the time of axillary dissection [64]. Using supermicrosurgery, arm lymphatics are connected with a collateral branch of the axillary vein distal to a competent valve [16, 65]. Among 46 women with breast cancer undergoing ALND randomized to no preventative surgical approach or to LYMPHA, lymphedema had occurred in 4.3% and 30.4% of the LYMPHA and control groups, respectively (*p* < 0.05) at 18-months [66].

While LYMPHA may be a promising technique, expertise in microsurgery, coordination between breast and plastic surgeons, and an upfront decision before surgery are required [67]. Additionally, there is risk associated with LYMPHA and a surgical learning curve. A 2021 study reported that 85% of breast surgeons reported not offering LYMPHA [68]. A simplified version of LYMPHA not requiring microsurgery has been described (SLYMPHA), with the procedure lowering BCRL incidence from 32 to 16% in one study [69]. Further surgical development for addressing lymphatic impairments is warranted along with algorithms for identifying best surgical candidates for the various interventions along with long-term surgical outcomes.

## Treatment of BCRL

**Fig. 1:**
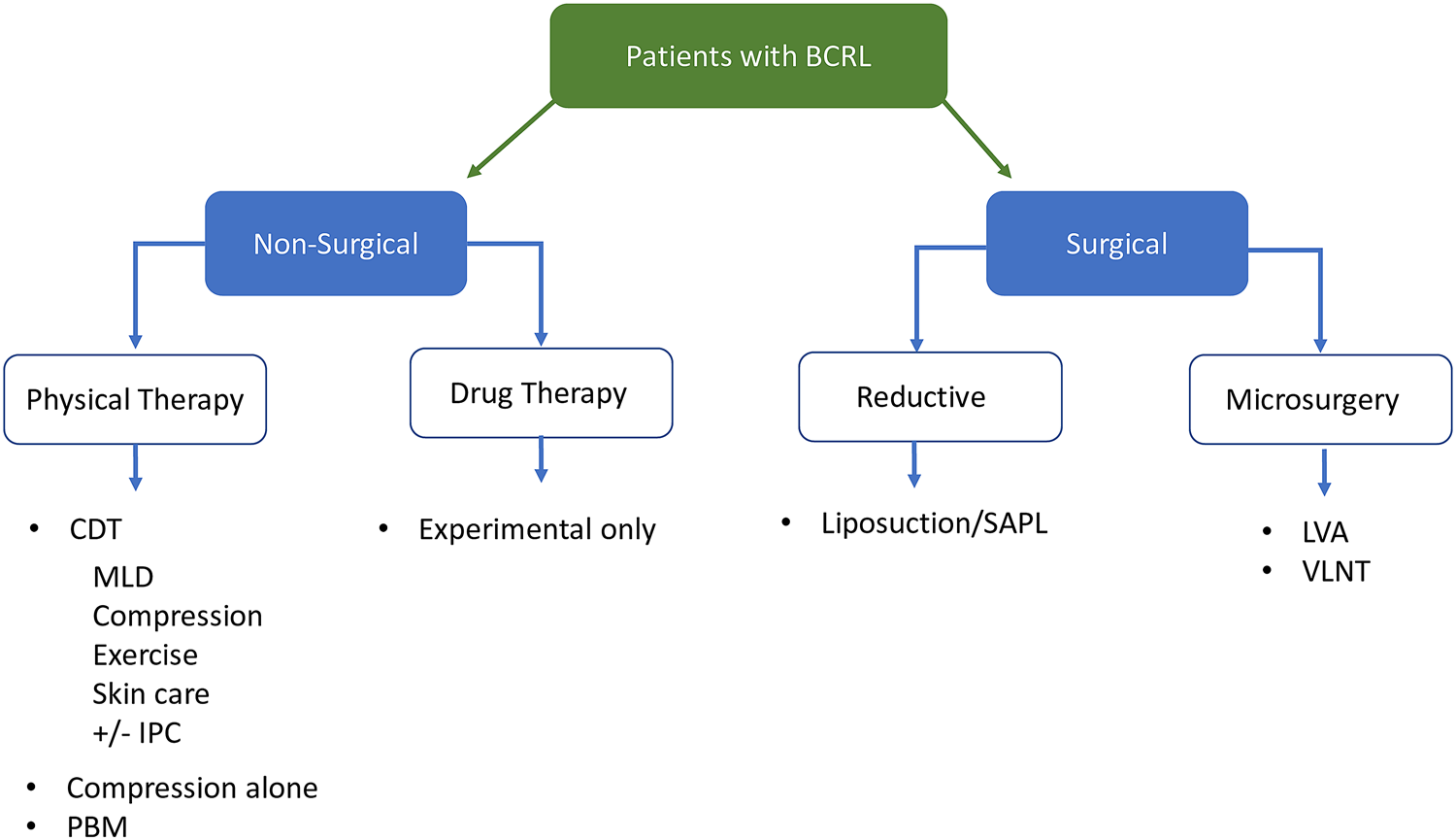
Major approaches to BCRL management. *CDT* complete decongestive therapy, *IPC* intermittent pneumatic compression, *LLLT* low-level laser therapy, *LVA* lymphovenous anastomosis, *MLD* manual lymphatic drainage, *SAPL* suction-assisted protein lipectomy, *VLNT* vascularized lymph node transfer

There is currently no approved drug therapy for lymphedema [70]. Recent clinical trials of pharmacological agents for the treatment of BCRL are summarized in Online Resource 1: Supplementary Table 3. Approaches to BCRL management are outlined in Fig. 1 and discussed below.

### Complete decongestive therapy (CDT)

The standard of care for patients with BCRL is Complete Decongestive Therapy (CDT, Complex Decongestive Therapy, Combined Physical Therapy [CPT], Complex Decongestive Physiotherapy [CDP], or Complex Lymphoedema Treatment [CLT]) [71, 72]. This multicomponent, multidisciplinary approach consists of an intensive treatment phase followed by a maintenance phase [73, 74].

The treatment phase focuses on MLD, multilayer short-stretch compression bandage (CB) wrapping and/or Velcro or other adjustable wraps, exercise to improve lymph flow [75], and meticulous skin care of the affected area [72]. MLD appears to stimulate lympholymphatic or lymphovenous anastomoses (LVA) [76]. The ability of MLD to facilitate transit of lymphatic fluids has been demonstrated using ICG fluorescence lymphography [77], potentially allowing for personalized MLD treatment [78]. Phase 1 of CDT is performed/supervised by a licensed clinician, typically with a physical or occupational degree, with specialized lymphedema training with a focus on maximal volume reduction and patient training [79]. Subsequently, patients are transitioned into their long-term maintenance phase (Phase 2) which involves self-care management of their chronic lymphedema using day and possibly night time compression, exercise, skin care, home pneumatic compression pumps and self lymphatic massage, to name a few, to maintain their optimal decongested state [79]. Resistance exercise has proven safe and demonstrated arm volume reduction benefits [80, 81]. Lymphedema maintenance being life-long, adherence to this complex, multi-modal regimen becomes problematic for many breast cancer survivors [82, 83].

There is little evidence that MLD alone is effective in BCRL management. A 2010 review of 16 trials found no consensus on the effectiveness of MLD alone [76]. In practice, however, MLD is used primarily as just one component of CDT. In that setting some [84, 85], but not all [86–90] analyses suggest that MLD may contribute to CDT benefit. In a recent systematic review and meta-analysis of 11 randomized controlled trials the addition of MLD to control treatments was associated with significant (*P* = 0.02) improvements in pain intensity but not arm volume reductions or QoL [90]. In a recent trial randomizing patients to fluorescence-guided MLD, normal MLD, or placebo MLD (all in combination with standardized CDT), all 3 groups had similar improvements in fluid accumulation and skin elasticity [91].

It is important to note that BCRL may affect the upper quadrant and/or the arm, thus therapy (including compression) addresses the area(s) of impairment. During the initial CDT phase, bandaging is applied to the limb and/or upper quadrant immediately after MLD treatment. The multilayer bandage, removed only for washing and MLD, applies a resting pressure during limb relaxation and a working pressure upon muscle contraction, mechanically stimulating the smooth muscle of lymph vessels [92]. Distinct from this, during the life-long maintenance phase of CDT, patients are fitted with compression garments to maintain the volume reduction achieved initially. These are not expected to provide any additional volume reductions yet are necessary for lymphedema containment and need to be properly fitted by a specialist. Compression garments provide transverse and longitudinal stretch with a high-to-low pressure gradient from above the wrist to the upper arm [93]. A full-sleeve compression garment is usually worn, sometimes with a glove to prevent dermal backflow. Compression garments must be replaced frequently, which adds to the financial burden as some insurers (e.g., Medicare) do not cover costs of compression garments (except compression bras), [94] though efforts are underway to improve insurance coverage.

Although CDT is regarded as the cornerstone of BCRL therapy [18, 65], evidence for its effectiveness varies [95]. CDT was found to be effective in reducing lymphedema in a systematic review of lymphedema studies from 2004 to 2011, although levels of evidence were only moderately strong [72, 96]. A 2007 retrospective analysis of 250 breast cancer survivors treated with CDT (55%), MLD (32%) or a home program (13%) agreed that these methods were collectively effective, with a mean 47% lymphedema volume reduction at 1 year (*p* < 0.0001) [97]. Not all studies, however, support the value of CDT relative to other, less resource-intensive treatments in BCRL. In a small randomized non-inferiority trial, compression bandaging plus exercise provided similar arm volume reductions and QoL improvements as CDT in post-mastectomy patients with arm edema [88].

A 2013 randomized trial compared elastic compression garments consisting of sleeve (30–40 mmHg) and glove alone (the control group) with CDT (intervention group) [98]. Mean excess arm volume reductions were 29.0% and 22.6%, respectively (*P* = 0.34) and QoL was similar in both groups. The trial was unable to demonstrate a significant improvement with CDT relative to compression garments, a surprising result given that compression garments are intended for containment and are not designed to enhance lymphatic pumping [10] warranting further investigation on this more simplified intervention and ideal patient candidate algorithm.

CDT is contraindicated in several conditions. Relative contraindications include uncontrolled hypertension, paralysis, diabetes, and bronchial asthma, while absolute contraindications include acute infections, uncontrolled congestive heart failure, and deep vein thrombosis [92]. Although it has been postulated that CDT/MLD might mobilize dormant tumor cells, thereby promoting cancer metastasis [92], studies suggest that this is not the case and CDT should not be withheld from patients with metastatic cancer [99].

Another compression method, intermittent pneumatic compression (IPC) as an adjunct to CDT was associated with additional mean volume reductions when used in either the initial treatment or the maintenance phase [100]. A 2022 systematic review concluded that based on existing evidence, IPC may provide an acceptable home-based treatment modality in addition to wearing compression garments in select patients with lymphedema [101]. Finally, a novel nonpneumatic active compression device (NPCD) that does not require patients to be immobile during use was recently evaluated in a randomized crossover trial [102]. Although results were encouraging, as with IPC, further studies are needed.

### Photobiomodulation (PBM)

Photobiomodulation (PBM), also known as low-level laser therapy (LLLT), is a type of phototherapy that uses light of wavelengths between 650 and 1000 nm delivered at low irradiance to the target site [103]. PBM has been shown to reduce inflammation, promote lymphatic mobility and regeneration, and prevent/manage fibrosis [103, 104]. Studies have examined PBM outcomes including arm volume/circumference, symptoms, and QoL [105–109]. Systematic reviews and meta-analyses have differed in their conclusions regarding its effectiveness in patients with BCRL [103, 110–112]. Larger randomized trials employing standardized protocols for treatment and assessment may clarify its potential benefit in this patient population, particularly when used in combination with CDT.

### Surgical treatment of BCRL

Although not necessarily with curative potential, advances in surgical approaches to BCRL treatment are likely to modify the current practice of typically reserving them for patients with lymphedema refractory to more conservative methods. Ultimately, the surgeries may go hand in hand with other conservative approaches. Lymphedema surgeries aim to either restore physiological lymphatic drainage ("reconstructive") or directly remove excess mass ("reductive").

### Reconstructive surgeries

LVA is a method of diverting lymph into the venous system, bypassing proximal obstruction. Lymphatic channels are identified, typically using ICG fluorescence imaging, a suitable recipient vein is also identified, and supermicrosurgical techniques are used to create an anastomosis between the two [113]. In patients with BCRL, studies have associated LVA with symptom improvement, arm volume reduction, and, notably, fewer episodes of cellulitis [113, 114]. After recovery, patients are urged to continue their previous therapies and wear compression garments [114]. More recently, however, a study of LVA side-to-end anastomoses in early grade lymphedema reported that it eliminated the need for compression garments later [115]. LVA is not curative, requires a multidisciplinary approach to integrate operative and post-operative management, and is technically demanding, requiring ICG fluorescence, supermicrosurgery instruments, and surgeons proficient in this specialized technique.

Rather than bypass obstructions in existing lymph node drainage, in vascular lymph node transfer (VLNT) an autologous lymph node flap microsurgically harvested from a distant donor site is transplanted to the target area with its blood supply preserved by anastomosing artery and vein in the graft to vessels at the receptor site, which may be axilla, elbow, or wrist. [116]. Donor sites include jejunal mesenteric, groin, lateral thoracic, omental, and submental [117]. Although the mechanism(s) by which lymphatic flow is restored is incompletely understood [118], improved lymphatic transport that results has been demonstrated in numerous studies [65].

A recent study examined outcomes for patients with Stage 2 primary or secondary lymphedema treated with pre-operative conservative therapy followed by VLNT [117]. Two years after surgery significant reductions in limb volume (mean 45.7%; *P* = 0.002), BIS scores (mean 59.8%; *P* < 0.001), and cellulitis episodes (97.9%; *P* < 0.001) were observed, and patient QoL per Lymphedema Life Impact Scale score was improved (mean 61.6%; *P* < 0.001). Complication rates were low.

**Fig. 2:**
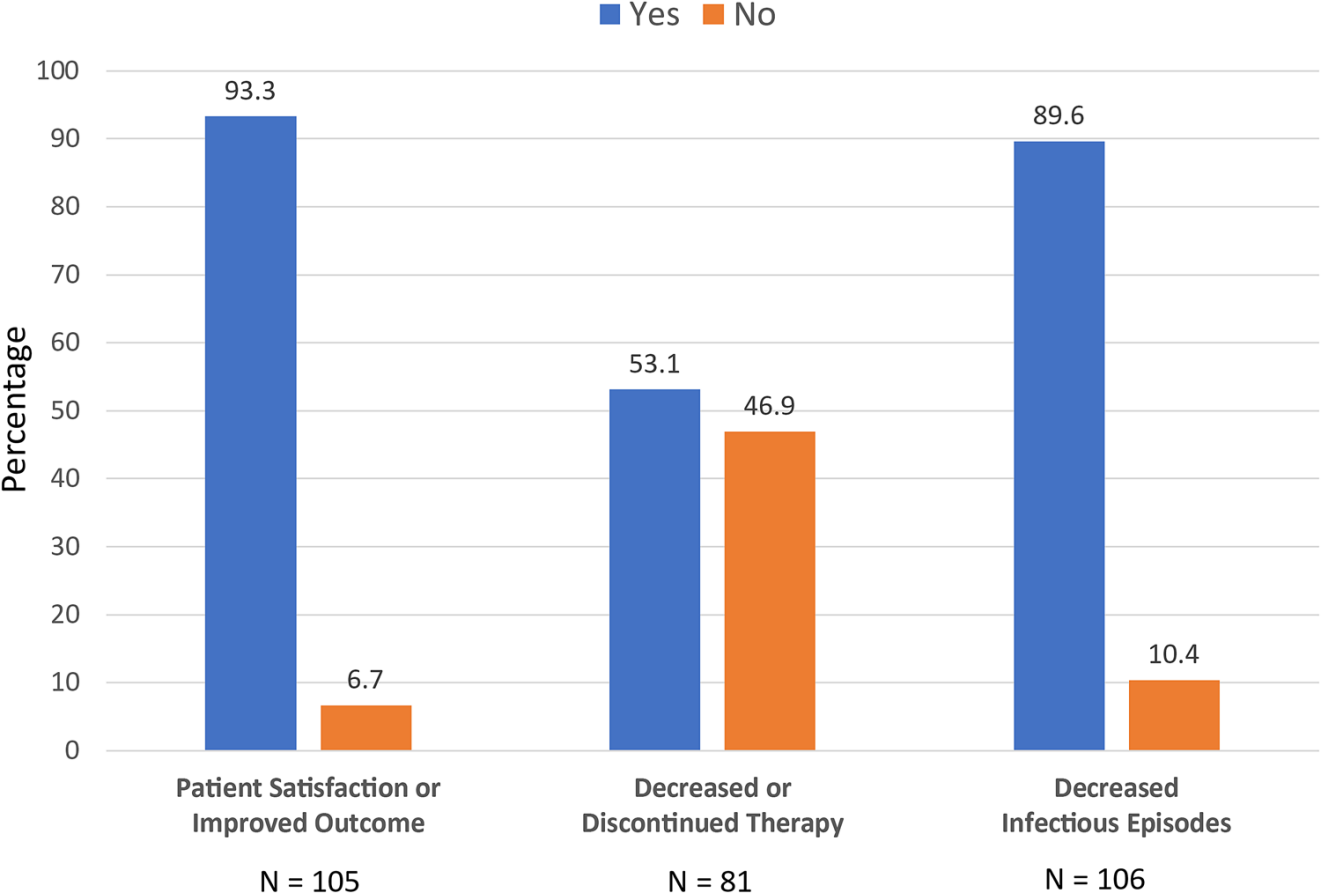
Reported outcomes in patients with BCRL after VLNT surgery. From a systematic review by Ozturk et al. [119]

Systematic reviews and meta-analyses have supported the benefits of VLNT in patients with lymphedema [119, 120]. In one such study, among patients who underwent lymphoscintigraphy or lymphangiography, 60% demonstrated moderate or significant flow improvement, and 93% reported a high satisfaction level (Fig. 2) [119]. Reports have suggested that VLNT may allow some patients to later reduce or eliminate conservative measures such as compression garment usage [120, 121], thereby ameliorating an economic burden and source of diminished QoL [122, 123].

Disadvantages of VLNT include risk of iatrogenic lymphedema at the donor site [124]. In a report of patients with secondary upper limb lymphedema undergoing VLNT at a specialized lymphology center, complication rates were 14.3% for irreversible lower limb lymphedema, 21.4% for lymphocele, and 14.3% for donor site pain [124]. Considering that patients likely undergo VLNT in the hope of curing their lymphedema, improvements in surgical techniques and patient identification will be essential for optimizing outcomes. Notably, VLNT changes to lymphatic function are gradual, with months or years needed to achieve full benefit [65], highlighting the importance of patient education in surgical expectations. Further advances in surgical technique(s), timing of surgical intervention (i.e. preventative or management of lymphatic impairment), and improved patient identification will be important in making treatment decisions regarding the role of reconstructive surgeries in BCRL.

### Reductive surgeries

In chronic, advanced stage lymphedema a high content of adipose and fibrotic tissues are present [125, 126]. Liposuction-based procedures such as suction assisted protein lipectomy (SAPL) can be used to remove excess solid volume in a lymphedema-affected arm [118, 127]. These techniques are generally reserved for patients with chronic, non-pitting BCRL [125] and do not restore lymphatic function, but reduce limb size for physical functioning improvement, easier daily self-management and more optimal quality of life. Edema volume reductions are rapid, with few complications reported, although it essential to underscore these surgical outcomes are from surgeons highly experienced in this specialized technique [118]. Mean percentage reductions in arm volume of 101–118% at 1–3 years after surgery are typically reported [128–130], and are long-lasting [129]. However, the underlying lymphatic impairment is not cured, and the involved region be maintained by constant, life-long compression garment usage [125]. A decreased incidence of infections and improved QoL have been associated with liposuction/SAPL in patients with BCRL [131, 132].

## Conclusions

BCRL affects more than one million breast cancer survivors worldwide. Breast cancer survivors face a lifelong risk of BCRL occurrence. It is generally incurable, negatively affects QoL, physical function, and daily activities, and requires lifelong management [56, 92, 133]. Its continuing burden (e.g., wearing expensive compression garments, avoidance of cuts and scratches) ultimately makes for low adherence, enabling more rapid progression and further disability. BCRL screening and education in at-risk patients are imperative, and an individualized approach to goal setting is recommended to improve adherence. Many patients with breast cancer report never having received information about BCRL, however [52], an unacceptable situation that needs to be remedied. Methods such as ARM and LYMPHA at the time of surgery have been shown to be effective in reducing BCRL incidence but come with their own risks and are not always feasible. Newer surgical methods for immediate lymphatic reconstruction will likely play an increasingly important role in BCRL prevention [69, 134, 135].

For breast cancer survivors with BCRL, CDT remains the current standard of care [71, 72]. It comes with high financial, time, and adherence requirements, and is not curative. Novel techniques such as IPC, PBM, and NPCD may have a place in BCRL treatment, but further studies are needed.

Surgical approaches to BCRL treatment continue to emerge with the intention of restoring normal lymphatic flow in patients with lymphedema. LVA and VLNT are two types of reconstructive surgery. Although often effective, they require specialized microsurgical or supermicrosurgical expertise and neither is curative. As these techniques continue to evolve, they may increasingly be used at an earlier stage in selected patients. Reductive surgery via liposuction/SAPL, performed by surgeons experienced in this specialized technique results in immediate volume reduction, but maintaining the new equilibrium requires the constant use of compression garments. Currently no drug has proven safe and effective in treating BCRL.

Assessing the impact of BCRL prophylaxis or treatment requires a comprehensive evaluation of patient- and clinician-reported outcomes. Perhaps the greatest barrier to progress in the prevention and treatment of BCRL is the current lack of standardized measures by which these outcomes can be compared. Progress in optimizing BCRL care must therefore encompass advances in patient education and investigator consensus as well as clinical techniques.

## Supplementary Information

Below is the link to the electronic supplementary material.

Supplementary file1 (PDF 163 KB)

## Data Availability

Not applicable.
