# Supplementary material for: Advances in the prevention and treatment of breast cancer-related lymphedema
Source: Breast Cancer Res Treat. 2023 Apr 27;200(1):1–14. doi: 10.1007/s10549-023-06947-7 (PMC10224871; doi:10.1007/s10549-023-06947-7)
Supplement: Supplementary file 1 — Supplementary file1 (PDF 163 KB) [file 10549_2023_6947_MOESM1_ESM.pdf]

ONLINE RESOURCE 1: SUPPLEMENTARY TABLES

Supplementary Table 1. Treatment-related risk factors for breast cancer-related lymphedema. Only selected prospective studies of >500 patients and meta-analyses are shown.

Risk Factor	Reference	Study Type	N	Follow-Up	Magnitude	*P*-value
ALND vs. SLND	DiSipio et al.[1]	Meta-analysis	--	3 - 360 mo	RR 2.7	--
	Ferguson et al.[2]	Prospective	632	m 24 mo	--	<0.001
	Lucci et al.[3]	Prospective	891	12 mo	13% vs. 2%	≤0.0001
	Mansel et al.[4]	Prospective	1031	12 mo	13% vs. 5%	--
	McDuff et al.[5]	Prospective	2171	48 mo	HR 2.71	<0.0001
	Miller et al.[6]	Prospective	616	22.2 mo	HR 7.53[a]	<0.0001
	Naoum et al.[7]	Prospective	1815	52.7 mo	24.9% vs. 8%	--
	Norman et al.[8]	Population-based	631	60 mo	HR 2.61[b]	--
	Swaroop et al.[9]	Prospective	1121	39.7 mo	HR 8.19	<0.0001
	Tsai et al.[10]	Meta-analysis	--	--	RR 3.07[b]	<0.0001
	Wetzig et al.[11]	Prospective	1088	60 mo	OR 0.22[c]	0.004
Greater no. LN dissected	DiSipio et al.[1]	Meta-analysis	NS	3 - 360 mo	HR 1.2	--
>10 Versus ≤3 positive LNs	Bundred et al.[12]	Prospective	1100	36 mo	OR 3.05	<0.001
Radiotherapy	DiSipio et al.[1]	Meta-analysis	--	3 - 360 mo	HR 1.3	--
	Ferguson et al.[2]	Prospective	632	m 24 mo	--	0.0364[d]
	Tsai et al.[10]	Meta-analysis	--	--	RR 1.92	<0.0001
	Whelan et al.[13]	Prospective	1832	9.5 yr	8.4% vs. 4.5%	0.0001[e]
Mastectomy vs. lumpectomy	DiSipio et al.[1]	Meta-analysis	--	3 - 360 mo	OR 2.7 - 7.4	--
	Tsai et al.[10]	Meta-analysis	--	--	RR 1.42	<0.0001
Immediate vs. no reconstruction	Miller et al.[6]	Prospective	616	22.2 mo	HR 0.432	<0.0001
Chemotherapy	Bevilacqua et al.[14]	Prospective	1054	41 mo	--	<0.0001[f]
	Bundred et al.[12]	Prospective	1100	36 mo	OR 1.57 [g]	0.008
	DiSipio et al.[1]	Meta-analysis	--	3 - 360 mo	HR 1.4 - 3.7	--
	Norman et al.[8]	Population-based	631	60 mo	HR 1.46[h]	--
	Swaroop et al.[9]	Prospective	1121	39.7 mo	HR 1.14[g]	0.62

ALND, axillary lymph node dissection; HR, hazard ratio; m, median; LN, lymph node; N, number of patients; OR, odds ratio; RR, risk ratio; SLND, sentinel lymph node dissection.

[a] ALND vs. SLNB/no axillary surgery.[b] ALND vs. no lymph node surgery.[c] SLNB vs ALND.[d] Regional LN radiation. [e] Whole-breast irradiation plus regional lymph node irradiation vs. whole-breast irradiation alone. [f] Number of cycles of adjuvant or neoadjuvant chemotherapy.[g] Adjuvant taxane chemotherapy. [h] Anthracycline-based chemotherapy.

This Supplementary Online Resource is from Advances in the Prevention and Treatment of Breast Cancer-Related Lymphedema in *Breast Cancer Research and Treatment* by Paula MC Donahue, Adrien MacKenzie, Aleksandra Filipovic and Louise Koelmeyer. Corresponding author email: paula.m.donahue@vumc.org

Supplementary Table 2. Summary of selected studies of non-treatment-related risk factors for breast cancer-related lymphedema.

Risk Factor	Reference	Study Type	N	Follow-Up	Magnitude	*P*-Value
Age	Bevilacqua et al.[14]	Prospective	1054	41 mo	--	0.0040
	Coriddi et al.[15]	Retrospective	1106	--	SC 9.65[a]	0.045
	Swaroop et al.[9]	Prospective	1121	39.7 mo	HR 1.02[b]	0.0433
Baseline BMI	Bevilacqua et al.[14]	Prospective	1054	41 mo	--	<0.0001
	DiSipio et al.[1]	Meta-analysis	NS	3 - 360 mo	RR 5.5	--
	Ferguson et al.[2]	Prospective	632	24 mo	--	0.0236[c]
	McDuff et al.[5]	Prospective	2171	48 mo	AHR 2.07	<0.0001
	McLaughlin et al.[16]	Prospective	936	60 mo	--	<0.0001
	Miller et al.[6]	Prospective	616	22.2 mo	HR 3.42[d]	<0.0001
	Swaroop et al.[9]	Prospective	1121	39.7 mo	HR 1.05[e]	0.0007
	Wetzig et al.[11]	Prospective	1088	60 mo	OR 1.15[e]	<0.001
Genetic factors	Miaskowski et al.[17]	Retrospective	542	--	--	<0.05[f]
Infection	Ferguson et al.[2]	Prospective	632	24 mo	--	<0.001[g]
	McLaughlin et al.[16]	Prospective	936	60 mo	--	<0.001
Race/ethnicity	Barrio et al.[18]	Prospective	268	18 mo	OR 4.41[h]	<0.001
	Kwan et al.[19]	Prospective	997	20.9 mo	HR 1.93 [h]	--
Subclinical edema	Bucci et al.[20]	Prospective	1790	--<br>--	HR 2.60[i]<br>HR 6.75[j]	0.0002[i]<br><0.0001[j]
	Bundred et al.[12]	Prospective	1100	36 mo	OR 6.60[k]	<0.001

AHR, adjusted hazard ratio; BMI, body mass index; RR, relative risk; SC, severity coefficient.
[a] Severity coefficient for risk in patients age ≥50 years.
[b] Hazard ratio per 1-year increment.
[c] For BMI ≥25 lb/in^2
[d] For BMI ≥30 kg/m^2
[e] With BMI as a continuous variable.
[f] Significant associations with 4 genes and three haplotypes involved in lymphangiogenesis and angiogenesis.
[g] For cellulitis.
[h] Among Black patients compared with Caucasian patients.
[i] Among patients with axial lymph node dissection with subclinical edema <3 months post-surgery.
[j] Among patients with sentinel lymph node dissection with subclinical edema <3 months post-surgery.
[k] Among patients with relative arm volume increase >10% 1-month post-surgery.

Supplementary Table 3. Recent interventional clinical trials of pharmacological agents for the treatment of breast cancer-related lymphedema.

Trial	Agent	Phase	N	Design	Primary Outcome	Results
NCT02257970 [21]	Ketoprofen	4	89	Open label exploratory, then randomized, double-blind, placebo-controlled trial of pts with arm or leg LE	Exploratory Phase: Derm. score<br>Randomized Phase: Skin thickness	Exploratory Phase:<br>Score improvement -3.4 ($p <$ 0.0001)<br>Randomized Phase:<br>Skin thickness reduced ($P =$ 0.01), but no change in limb volume
NCT04243837 [22]	LYT-100	1/2	100	Part 1: Dose escalation<br>Part 2: Food effect study<br>Part 3: Randomized, double-blind, placebo-controlled trial in pts with Stage 1 or 2 BCRL	Part 1: safety, MTD<br>Part 2: PK, food effect<br>Part 3: Efficacy signals	Part 1: well tolerated, MTD not reached at 100 - 1000 mg BID<br>Part 2: 19% lower exposure in fed vs. fasting<br>Part 3: Results not yet reported
NCT02994771 [23]	Lymfactin®	1	15	Single-arm trial of adenoviral VEGF-C combined with VLNT in pts with BCRL	Safety	Well tolerated, no DLT at maximum dose
NCT03658967 [24]	Lymfactin®	2	39	Double-blind, randomized, placebo-controlled trial of adenoviral VEGF-C combined with VLNT in pts with BCRL	Arm volume; Lymphatic flow by lymphoscintigraphy; QoL per LQOLI	Company press release states that results were "inconclusive"[25]
NCT04390685	Tacrolimus, topical	1/2	60	Single-arm study of tacrolimus ointment following ALND for BCRL prevention	Arm volume change by water displacement	Not yet reported
NCT04541290	Tacrolimus, topical	1/2	20	Single-arm study of tacrolimus ointment in pts with existing BCRL	Arm volume change by water displacement	Not yet reported
NCT02494206[26]	QBX258	NS	9	Single-arm study of anti-IL4/ anti-IL13 blockade in Stage 1 or 2 BCRL	Arm volume change by perometry	Arm volume significantly *increased* relative to baseline (p = 0.046)

ALND, axillary lymph node dissection; BCRL, breast cancer-related lymphedema; BID, twice-daily; LE, lymphedema; LQLOLI, lymphedema quality of life inventory; MTD, maximum tolerated dose; NS, not specified; QoL, quality of life; VEGF-C, vascular endothelial growth factor C; VLNT, vascularized lymph node transfer

This Supplementary Online Resource is from Advances in the Prevention and Treatment of Breast Cancer-Related Lymphedema in *Breast Cancer Research and Treatment* by Paula MC Donahue, Adrien MacKenzie, Aleksandra Filipovic and Louise Koelmeyer. Corresponding author email: paula.m.donahue@vumc.org

SUPPLEMENTARY TABLE REFERENCES

1. DiSipio T, Rye S, Newman B, Hayes S (2013) Incidence of unilateral arm lymphoedema after breast cancer: a systematic review and meta-analysis. Lancet Oncol 14:500-515. doi: 10.1016/S1470-2045(13)70076-7
2. Ferguson CM, Swaroop MN, Horick N, Skolny MN, Miller CL, Jammallo LS, Brunelle C, O'Toole JA, Salama L, Specht MC, Taghian AG (2016) Impact of ipsilateral blood draws, injections, blood pressure measurements, and air travel on the risk of lymphedema for patients treated for breast cancer. J Clin Oncol 34:691-698. doi: 10.1200/JCO.2015.61.5948
3. Lucci A, McCall LM, Beitsch PD, Whitworth PW, Reintgen DS, Blumencranz PW, Leitch AM, Saha S, Hunt KK, Giuliano AE, American College of Surgeons Oncology G (2007) Surgical complications associated with sentinel lymph node dissection (SLND) plus axillary lymph node dissection compared with SLND alone in the American College of Surgeons Oncology Group Trial Z0011. J Clin Oncol 25:3657-3663. doi: 10.1200/JCO.2006.07.4062
4. Mansel RE, Fallowfield L, Kissin M, Goyal A, Newcombe RG, Dixon JM, Yiangou C, Horgan K, Bundred N, Monypenny I, England D, Sibbering M, Abdullah TI, Barr L, Chetty U, Sinnett DH, Fleissig A, Clarke D, Ell PJ (2006) Randomized multicenter trial of sentinel node biopsy versus standard axillary treatment in operable breast cancer: the ALMANAC Trial. J Natl Cancer Inst 98:599-609. doi: 10.1093/jnci/djj158
5. McDuff SGR, Mina AI, Brunelle CL, Salama L, Warren LEG, Abouegylah M, Swaroop M, Skolny MN, Asdourian M, Gillespie T, Daniell K, Sayegh HE, Naoum GE, Zheng H, Taghian AG (2019) Timing of lymphedema after treatment for breast cancer: when are patients most at risk? Int J Radiat Oncol Biol Phys 103:62-70. doi: 10.1016/j.ijrobp.2018.08.036
6. Miller CL, Colwell AS, Horick N, Skolny MN, Jammallo LS, O'Toole JA, Shenouda MN, Sadek BT, Swaroop MN, Ferguson CM, Smith BL, Specht MC, Taghian AG (2016) Immediate implant reconstruction is associated with a reduced risk of lymphedema compared to mastectomy alone: a prospective cohort study. Ann Surg 263:399-405. doi: 10.1097/SLA.0000000000001128
7. Naoum GE, Roberts S, Brunelle CL, Shui AM, Salama L, Daniell K, Gillespie T, Bucci L, Smith BL, Ho AY, Taghian AG (2020) Quantifying the impact of axillary surgery and nodal irradiation on breast cancer-related lymphedema and local tumor control: long-term results from a prospective screening trial. J Clin Oncol 38:3430-3438. doi: 10.1200/JCO.20.00459
8. Norman SA, Localio AR, Kallan MJ, Weber AL, Torpey HA, Potashnik SL, Miller LT, Fox KR, DeMichele A, Solin LJ (2010) Risk factors for lymphedema after breast cancer treatment. Cancer Epidemiol Biomarkers Prev 19:2734-2746. doi: 10.1158/1055-9965.EPI-09-1245
9. Swaroop MN, Ferguson CM, Horick NK, Skolny MN, Miller CL, Jammallo LS, Brunelle CL, O'Toole JA, Isakoff SJ, Specht MC, Taghian AG (2015) Impact of adjuvant taxane-based chemotherapy on development of breast cancer-related lymphedema: results from a large prospective cohort. Breast Cancer Res Treat 151:393-403. doi: 10.1007/s10549-015-3408-1
10. Tsai RJ, Dennis LK, Lynch CF, Snetselaar LG, Zamba GK, Scott-Conner C (2009) The risk of developing arm lymphedema among breast cancer survivors: a meta-analysis of treatment factors. Ann Surg Oncol 16:1959-1972. doi: 10.1245/s10434-009-0452-2
11. Wetzig N, Gill PG, Espinoza D, Mister R, Stockler MR, Gebski VJ, Ung OA, Campbell I, Simes J (2017) Sentinel-lymph-node-based management or routine axillary clearance?

five-year outcomes of the RACS Sentinel Node Biopsy Versus Axillary Clearance (SNAC) 1 Trial: assessment and incidence of true lymphedema. Ann Surg Oncol 24:1064-1070. doi: 10.1245/s10434-016-5669-2
12. Bundred N, Foden P, Todd C, Morris J, Watterson D, Purushotham A, Bramley M, Riches K, Hodgkiss T, Evans A, Skene A, Keeley V, Investigators of BEAPs (2020) Increases in arm volume predict lymphoedema and quality of life deficits after axillary surgery: a prospective cohort study. Br J Cancer 123:17-25. doi: 10.1038/s41416-020-0844-4
13. Whelan TJ, Olivotto IA, Parulekar WR, Ackerman I, Chua BH, Nabid A, Vallis KA, White JR, Rousseau P, Fortin A, Pierce LJ, Manchul L, Chafe S, Nolan MC, Craighead P, Bowen J, McCready DR, Pritchard KI, Gelmon K, Murray Y, Chapman JA, Chen BE, Levine MN, Investigators MAS (2015) Regional nodal irradiation in early-stage breast cancer. N Engl J Med 373:307-316. doi: 10.1056/NEJMoa1415340
14. Bevilacqua JL, Kattan MW, Changhong Y, Koifman S, Mattos IE, Koifman RJ, Bergmann A (2012) Nomograms for predicting the risk of arm lymphedema after axillary dissection in breast cancer. Ann Surg Oncol 19:2580-2589. doi: 10.1245/s10434-012-2290-x
15. Coriddi M, Khansa I, Stephens J, Miller M, Boehmler J, Tiwari P (2015) Analysis of factors contributing to severity of breast cancer-related lymphedema. Ann Plast Surg 74:22-25. doi: 10.1097/SAP.0b013e31828d7285
16. McLaughlin SA, Wright MJ, Morris KT, Giron GL, Sampson MR, Brockway JP, Hurley KE, Riedel ER, Van Zee KJ (2008) Prevalence of lymphedema in women with breast cancer 5 years after sentinel lymph node biopsy or axillary dissection: objective measurements. J Clin Oncol 26:5213-5219. doi: 10.1200/JCO.2008.16.3725
17. Miaskowski C, Dodd M, Paul SM, West C, Hamolsky D, Abrams G, Cooper BA, Elboim C, Neuhaus J, Schmidt BL, Smoot B, Aouizerat BE (2013) Lymphatic and angiogenic candidate genes predict the development of secondary lymphedema following breast cancer surgery. PLoS One 8:e60164. doi: 10.1371/journal.pone.0060164
18. Barrio AV, Montagna G, Sevilimedu V, Gomez EA, Mehrara B, Morrow M (2021) Impact of race and ethnicity on incidence and severity of breast cancer related lymphedema after axillary lymph node dissection: Results of a prospective screening study. Poster GS4-01 at San Antonio Breast Cancer Symposium, Dec 7-10. In:
19. Kwan ML, Darbinian J, Schmitz KH, Citron R, Partee P, Kutner SE, Kushi LH (2010) Risk factors for lymphedema in a prospective breast cancer survivorship study: the Pathways Study. Archives of surgery (Chicago, Ill. : 1960) 145:1055-1063. doi: 10.1001/archsurg.2010.231
20. Bucci LK, Brunelle CL, Bernstein MC, Shui AM, Gillespie TC, Roberts SA, Naoum GE, Taghian AG (2021) Subclinical lymphedema after treatment for breast cancer: risk of progression and considerations for early intervention. Ann Surg Oncol 28:8624-8633. doi: 10.1245/s10434-021-10173-0
21. Rockson SG, Tian W, Jiang X, Kuznetsova T, Haddad F, Zampell J, Mehrara B, Sampson JP, Roche L, Kim J, Nicolls MR (2018) Pilot studies demonstrate the potential benefits of antiinflammatory therapy in human lymphedema. JCI Insight 3. doi: 10.1172/jci.insight.123775
22. Chen MC, Korth CC, Harnett MD, Elenko E, Lickliter JD (2022) A randomized phase 1 evaluation of deupirfenidone, a novel deuterium-containing drug candidate for interstitial lung disease and other inflammatory and fibrotic diseases. Clin Pharmacol Drug Dev 11:220-234. doi: 10.1002/cpdd.1040
23. Hartiala P, Suominen S, Suominen E, Kaartinen I, Kiiski J, Viitanen T, Alitalo K, Saarikko AM (2020) Phase 1 Lymfactin(®) study: short-term safety of combined adenoviral VEGF-

C and lymph node transfer treatment for upper extremity lymphedema. J Plast Reconstr Aesthet Surg 73:1612-1621. doi: 10.1016/j.bjps.2020.05.009
24. Hartiala P, Lahdenperä O, Vuolanto A, Saarikko A (2020) Abstract OT1-06-01: Lymfactin, an investigational adenoviral gene therapy expressing VEGF-C, is currently studied in a double-blind, randomized, placebo-controlled, multicenter, phase 2 clinical study in patients suffering from breast cancer associated secondary lymphedema (BCAL). Cancer Research 80:OT1-06-01-OT01-06-01. doi: 10.1158/1538-7445.Sabcs19-ot1-06-01
25. Herantis Announces Inconclusive Results from Phase II Study with Lymfactin in Breast Cancer Related Lymphedema [press release]. March 2, 2021. https://herantis.com/press-releases/herantis-announces-inconclusive-results-from-phase-ii-study-with-lymfactin-in-breast-cancer-related-lymphedema/
26. Mehrara BJ, Park HJ, Kataru RP, Bromberg J, Coriddi M, Baik JE, Shin J, Li C, Cavalli MR, Encarnacion EM, Lee M, Van Zee KJ, Riedel E, Dayan JH (2021) Pilot study of anti-Th2 immunotherapy for the treatment of breast cancer-related upper extremity lymphedema. Biology (Basel) 10. doi: 10.3390/biology10090934
